# Supplementary material for: Land-use diversity predicts regional bird taxonomic and functional richness worldwide
Source: Nat Commun. 2023 Mar 10;14:1320. doi: 10.1038/s41467-023-37027-5 (PMC10006419; doi:10.1038/s41467-023-37027-5)
Supplement: Supplementary file 1 — Supplementary Information [file 41467_2023_37027_MOESM1_ESM.pdf]

Supplementary Material

Title: Land-use diversity predicts regional bird taxonomic and functional richness worldwide

Authors:

Carlos Martínez-Núñez[1,2,] *, Ricardo Martínez-Prentice[3], Vicente García-Navas[1,4]

Affiliations:

[1] Department of Integrative Ecology, Estación Biológica de Doñana EBD (CSIC), Seville, Spain.

[2] Agroscope. Reckenholzstrasse 191, CH-8046 Zurich. Switzerland.

[3] Institute of Agriculture and Environmental Sciences, Estonian University of Life Sciences, Tartu, Estonia.

[4] Department of Evolutionary Biology and Environmental Studies, University of Zurich, Zurich, Switzerland.

Corresponding author:

Carlos Martínez-Núñez, cmnunez@ujaen.es.

Supplementary Table 1: Traits pre-selected, their category and ecological importance. The ten categories of the trait trophic niche are: aquatic predator, frugivore, granivore, herbivore aquatic, herbivore terrestrial, invertivore, nectarivore, omnivore, scavenger and vertivore. The five categories of the trait primary lifestyle are: aerial, aquatic, generalist, insessorial and terrestrial.

Trait name	Trait type and levels	Trait category	Ecological Importance	Literature
**Body mass**	Quantitative (continuous)	*Size and Morphology*	Closely related to thermodynamics. It correlates with many different aspects of species biology	Dunning, 2007; Pigot et al. (2020)
**Length of tarsus**	Quantitative (continuous)	*Size and Morphology*	Strong influence on the foraging mode and provisioning behaviour. Affects locomotion	Fitzpatrick (1987)
**Tail length**	Quantitative (continuous)	*Size and Morphology*	Affects maneuverability and stability and thus, confers aerodynamic properties	
**Wing length**	Quantitative (continuous)	*Size and Morphology*	Affects flight efficiency	
**Hand-wing index**	Quantitative (continuous)	*Dispersal capacity*	Proxy of dispersal ability	Sheard et al. (2020)
**Length to the culmen**	Quantitative (continuous)	*Beak morphology*	Primary apparatus used by birds to capture and process food. It is associated to feeding interactions.	Grant (1999); Cooney et al. (2017); Navalón et al. (2019); Pigot et al. (2020)
**Length to the nares**	Quantitative (continuous)	*Beak morphology*		
**Beak width**	Quantitative (continuous)	*Beak morphology*		
**Beak depth**	Quantitative (continuous)	*Beak morphology*		
**Diet or predominant trophic niche**	Qualitative (factor; 10 levels)	*Trophic niche*	Determines which trophic resources (niche) are used by a particular species and thus, its role on the ecosystem	Felice et al. (2019)
**Primary lifestyle**	Qualitative (factor; 5 levels)	*Life form*	Informs about physiological and behavioral adaptations to specific ways of living, strata used, etc.	Wilman et al. (2014)

Supplementary Table 2: Habitat types (22 classes) from Copernicus landcover maps used to calculate the Shannon diversity index in each grid cell. Source: https://developers.google.com/earth-engine/datasets/catalog/COPERNICUS_Landcover_100m_Proba-V-C3_Global#bands.

Habitat class	Habitat description
1	Shrubs. Woody perennial plants with persistent and woody stems and without any defined main stem being less than 5 m tall. The shrub foliage can be either evergreen or deciduous.
2	Herbaceous vegetation. Plants without persistent stem or shoots above ground and lacking definite firm structure. Tree and shrub cover is less than 10 %.
3	Cultivated and managed vegetation / agriculture. Lands covered with temporary crops followed by harvest and a bare soil period (e.g., single and multiple cropping systems). Note that perennial woody crops will be classified as the appropriate forest or shrub land cover type.
4	Urban / built up. Land covered by buildings and other man-made structures.
5	Bare / sparse vegetation. Lands with exposed soil, sand, or rocks and never has more than 10 % vegetated cover during any time of the year.
6	Snow and ice. Lands under snow or ice cover throughout the year.
7	Permanent water bodies. Lakes, reservoirs, and rivers. Can be either fresh or salt-water bodies.
8	Herbaceous wetland. Lands with a permanent mixture of water and herbaceous or woody vegetation. The vegetation can be present in either salt, brackish, or fresh water.
9	Moss and lichen.
10	Closed forest, evergreen needle leaf. Tree canopy >70 %, almost all needle leaf trees remain green all year. Canopy is never without green foliage.
11	Closed forest, evergreen broad leaf. Tree canopy >70 %, almost all broadleaf trees remain green year-round. Canopy is never without green foliage.
12	Closed forest, deciduous needle leaf. Tree canopy >70 %, consists of seasonal needle leaf tree communities with an annual cycle of leaf-on and leaf-off periods.
13	Closed forest, deciduous broad leaf. Tree canopy >70 %, consists of seasonal broadleaf tree communities with an annual cycle of leaf-on and leaf-off periods.
14	Closed forest, mixed.
15	Closed forest, not matching any of the other definitions.

16	Open forest, evergreen needle leaf. Top layer- trees 15-70 % and second layer- mixed of shrubs and grassland, almost all needle leaf trees remain green all year. Canopy is never without green foliage.
17	Open forest, evergreen broad leaf. Top layer- trees 15-70 % and second layer- mixed of shrubs and grassland, almost all broadleaf trees remain green year-round. Canopy is never without green foliage.
18	Open forest, deciduous needle leaf. Top layer- trees 15-70 % and second layer- mixed of shrubs and grassland, consists of seasonal needle leaf tree communities with an annual cycle of leaf-on and leaf-off periods.
19	Open forest, deciduous broad leaf. Top layer- trees 15-70 % and second layer- mixed of shrubs and grassland, consists of seasonal broadleaf tree communities with an annual cycle of leaf-on and leaf-off periods.
20	Open forest, mixed.
21	Open forest, not matching any of the other definitions.
22	Oceans, seas. Can be either fresh or salt-water bodies.

Supplementary Table 3. Environmental data, sources, resolution calculated for each grid cell worldwide.

Environmental variable	Definition	Source	Resolution	References
Altitude	Digital Elevation Model. Topographic surface of the Earth excluding trees, buildings and any other surface objects (USGS)	WorldClim. Elevation data from Shuttle Radar Topography Mission (SRTM) and GTOPO30 data for areas north of 60N and south of 60S where SRTM was not available. We removed negative values.	2.5 minutes, derived from 30 arc second.	Processing of SRTM by WorldClim2 (Fick & Hijmans, 2017).
Percentage of land	Percent of inland (%) in each grid. No holes for lakes, thus, these are included as inland.	all_countries.shp	Vector format	
Temperature	Skin temperature (surface)	Netcdf file . Temporal cover: 2017 - 2018	Global gridded data at 0.1x0.1 degrees of latitude and longitude.	"ERA5-Land monthly averaged data from 1950 to present" stored in Climate Data Store, Copernicus service. https://cds.climate.copernicus.eu/. Official ERA5 documentation stored in ECMWF: https://confluence.ecmwf.int/
Precipitation	Total precipitation (m). Accumulated liquid and frozen water, including rain and snow, that falls to the Earth's surface. Units are depth in metres. It is the depth the water would have if it were spread evenly over the grid box	Netcdf file . Temporal cover: 2017 - 2018	Global gridded data at 0.1x0.1 degrees of latitude and longitude. (11.1 km)	"ERA5-Land monthly averaged data from 1950 to present" stored in Climate Data Store, Copernicus service. https://cds.climate.copernicus.eu/ Official ERA5 documentation stored in ECMWF: https://confluence.ecmwf.int/
Human Footprint	Dataset of annual dynamics of 8 different variables related to human pressure on the eco-environment, from 2000 to 2018. Pressure measured from 8 variables. Coverage: Earth's non-Antarctic terrestrial surface. For any grid cell, the Human Footprint can range between 0–50	hfp2018.tif	1 km resolution	Human Footprint dataset from 2000 to 2018 (Mu et al., 2022).<br><br>Data repository: https://figshare.com/articles/figure/An_annual_global_terrestrial_Human_Footprint_dataset_from_2000_to_2018/16571064
Realm	Division of Earth surface based on organisms. This is the broadest biogeographical division.	Ecoregions2017.shp	Vector format	Dinerstein et al., 2017.
Land-use diversity	Diversity of land-use pixels in a specific grid cell.	Calculation of Shannon-Wiener diversity index of habitats or land-use types in each grid cell using the Copernicus Global Land Service Land Cover Map (raster format)	100 m	Buchhorn et al., 2020; Gorelick et al., 2017; Magurran & Henderson, 2003.
Primary net productivity	Used NDVI (5-year Minimum, median, maximum, average, standard deviation and	Netcdf file . Temporal cover: 2015 - 2019	1 km	Copernicus Global Land Service website. PROBA-V Mission Exploitation Platform. Short term

	number of observations per 10-day) as an indicator of NPP (net primary production).<br>pv: physical value of NDVI: conversion factor from 0-255 values to -1 to 1: Transformation equation: https://land.copernicus.eu/global/products/ndvi			statistics. Baret et al., 2013; León-Tavares et al., 2021.

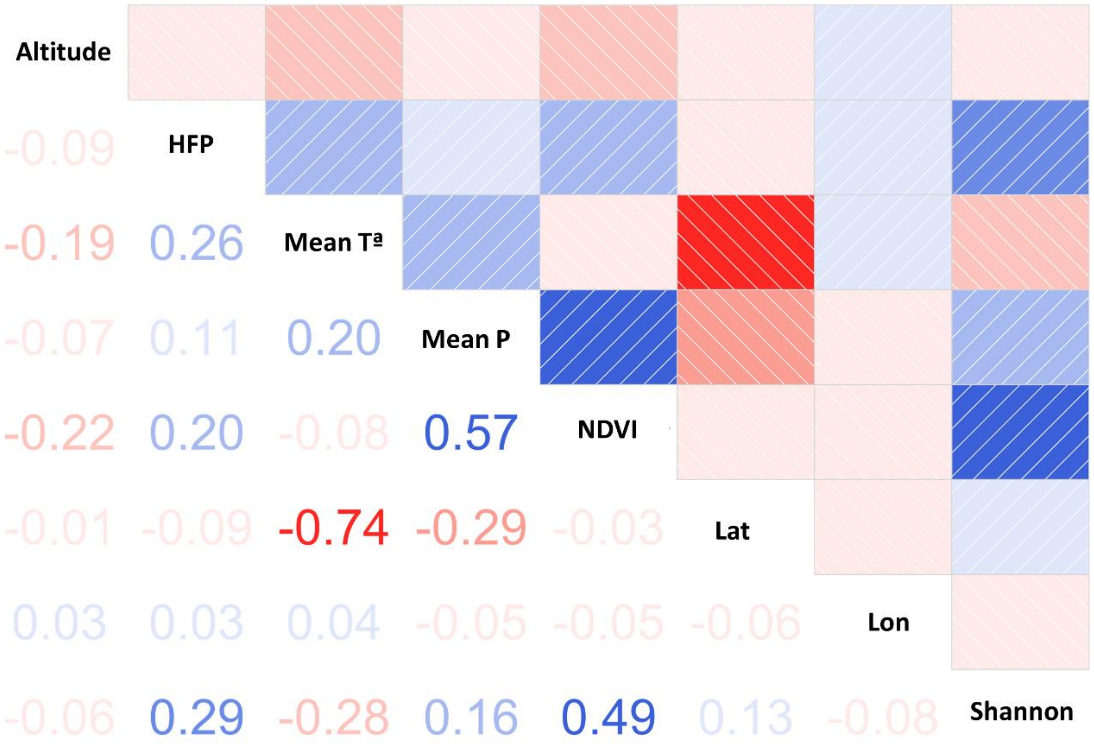

Supplementary Fig. 1. Pairwise correlations between potentially important explanatory variables. Potential macroecological drivers affecting the distribution and regional richness of bird assemblages: Altitude, human footprint (sensu Mu et al., 2022), mean annual temperature, mean precipitation, primary productivity measured through the NDVI, latitude (Lat), longitude (Lon) and Shannon index of land-use diversity based on vegetation cover maps (Shannon). The only potential confounding factor with Shannon land-use diversity is primary productivity (NDVI). N = 15,780 grid cells worldwide.

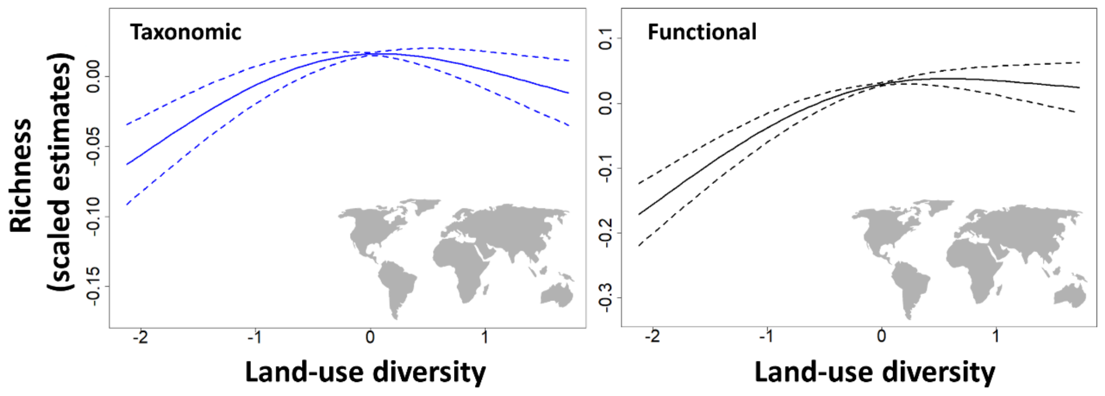

Supplementary Fig. 2. Partial effect of land-use diversity on regional taxonomic and functional richness across the world. Worldwide relationship (given a random biogeographic realm) between land-use diversity and bird regional taxonomic and functional richness, once accounted for the effects of latitude, longitude and primary productivity. Solid lines show predicted values and dashed lines show 95% CI. Note that estimated values in the y axis are scaled. N = 15,780 grid cells worldwide. World maps were obtained from https://freesvg.org.

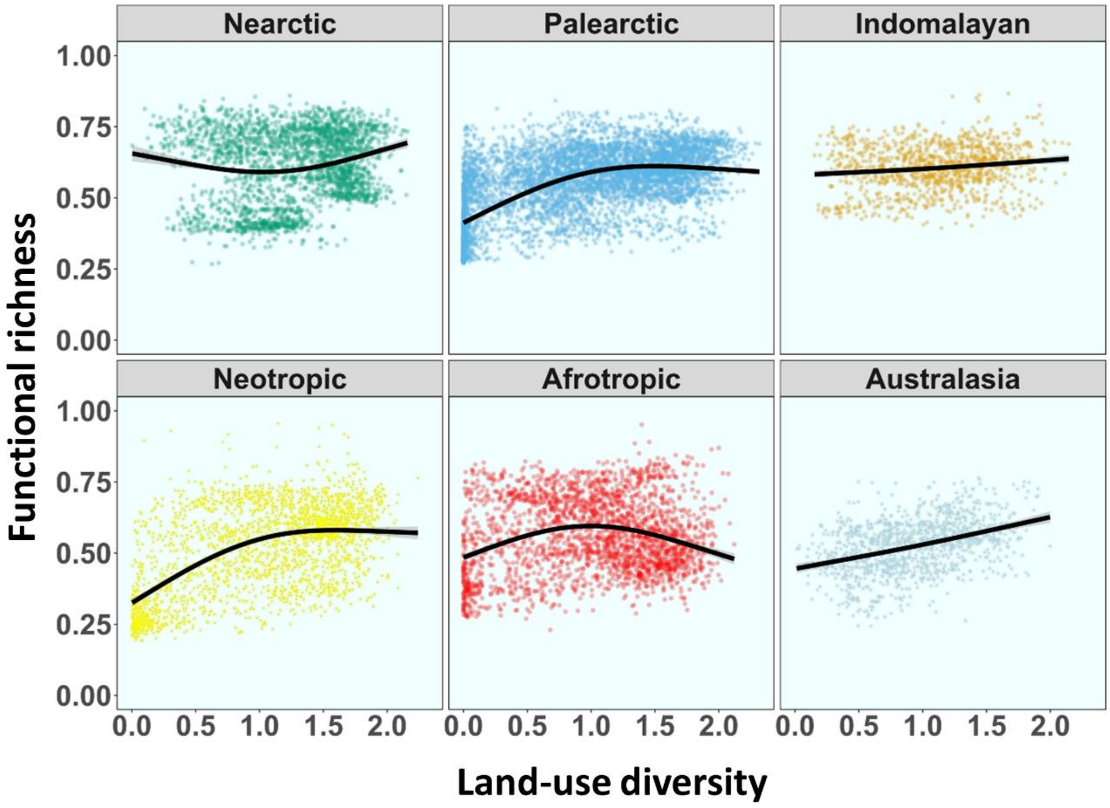

Supplementary Fig. 3. Change on bird regional functional richness due to land-use diversity, after accounting for taxonomic richness. Additive model fit (solid lines) to the partial residuals (points) of land-use diversity association with bird functional richness, after controlling for taxonomic richness. Taxonomic and functional richness were standardized to the maximum value observed. Solid line shows the model fit and line shadows represent 95% confidence intervals of the estimated fit. Nearctic: n= 2,630 grid cells. Neotropic: n = 2,262 grid cells. Palearctic: n= 6,123 grid cells. Afrotropic: n= 2,466 grid cells. Australasia: n= 1,212 grid cells. Indomalaya: n= 1,087 grid cells.

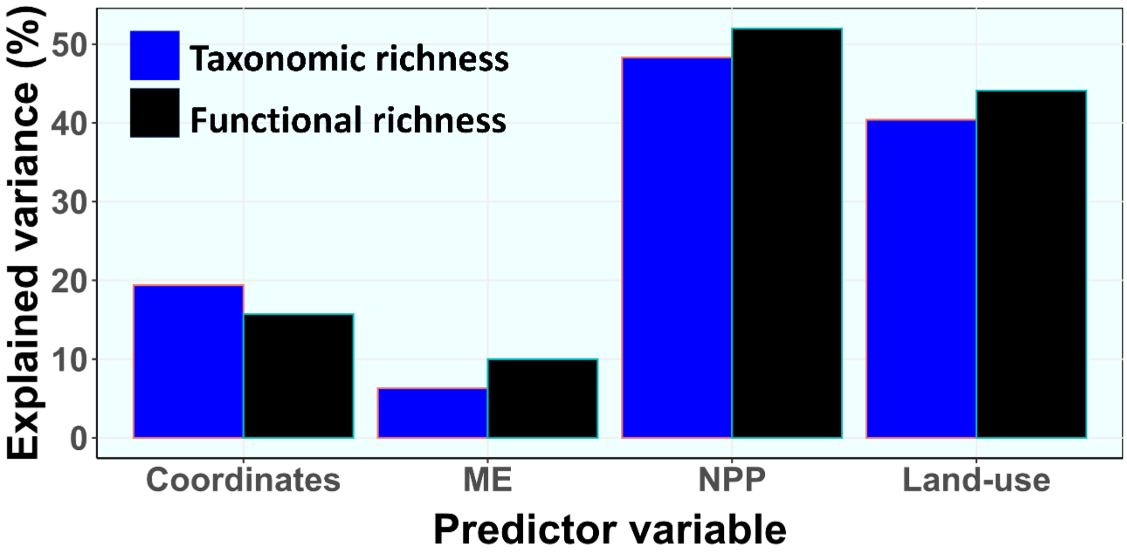

Supplementary Fig. 4. Relative contribution of predictors to explained variance. Deviance explained by models including each single variable separately in addition to coordinates (to control for spatial autocorrelation; variance explained by coordinates alone was subtracted). Note that coordinates were not included as an interaction term with biogeographic realm. ME=Median elevation. NPP = Net Primary Productivity (NDVI). Land-use = Shannon land-use diversity index. N = 15,780 grid cells worldwide.

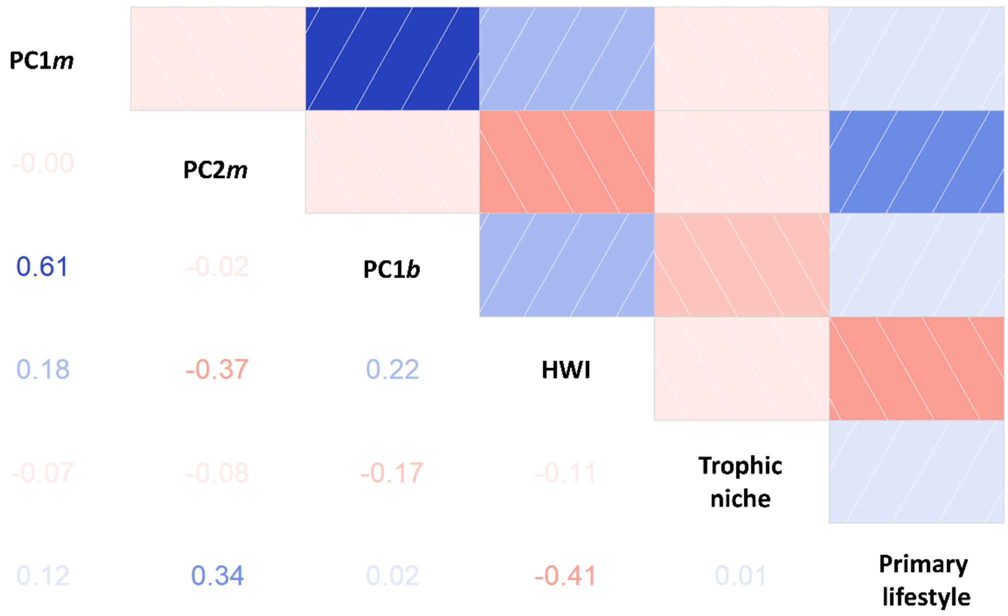

Supplementary Fig. 5. Correlogram showing pairwise correlation coefficients between the traits that were used to calculate the PCoA eigenvalues. PC1*m* = eigenvalues of the first principal component, based on morphological traits plus body mass. PC2*m* = eigenvalues of the second principal component, based on morphological traits plus body mass. PC1*b* = eigenvalues of the first principal component, based on beak traits previously corrected by body mass. HWI = Hand-wing index. Trophic niche = Categorical variable with 10 levels defining the predominant diet (transformed to numeric here). Primary lifestyle = Categorical variable (transformed to numeric here) defining the lifestyle of each bird species (e.g., aquatic, terrestrial, insessorial, etc).

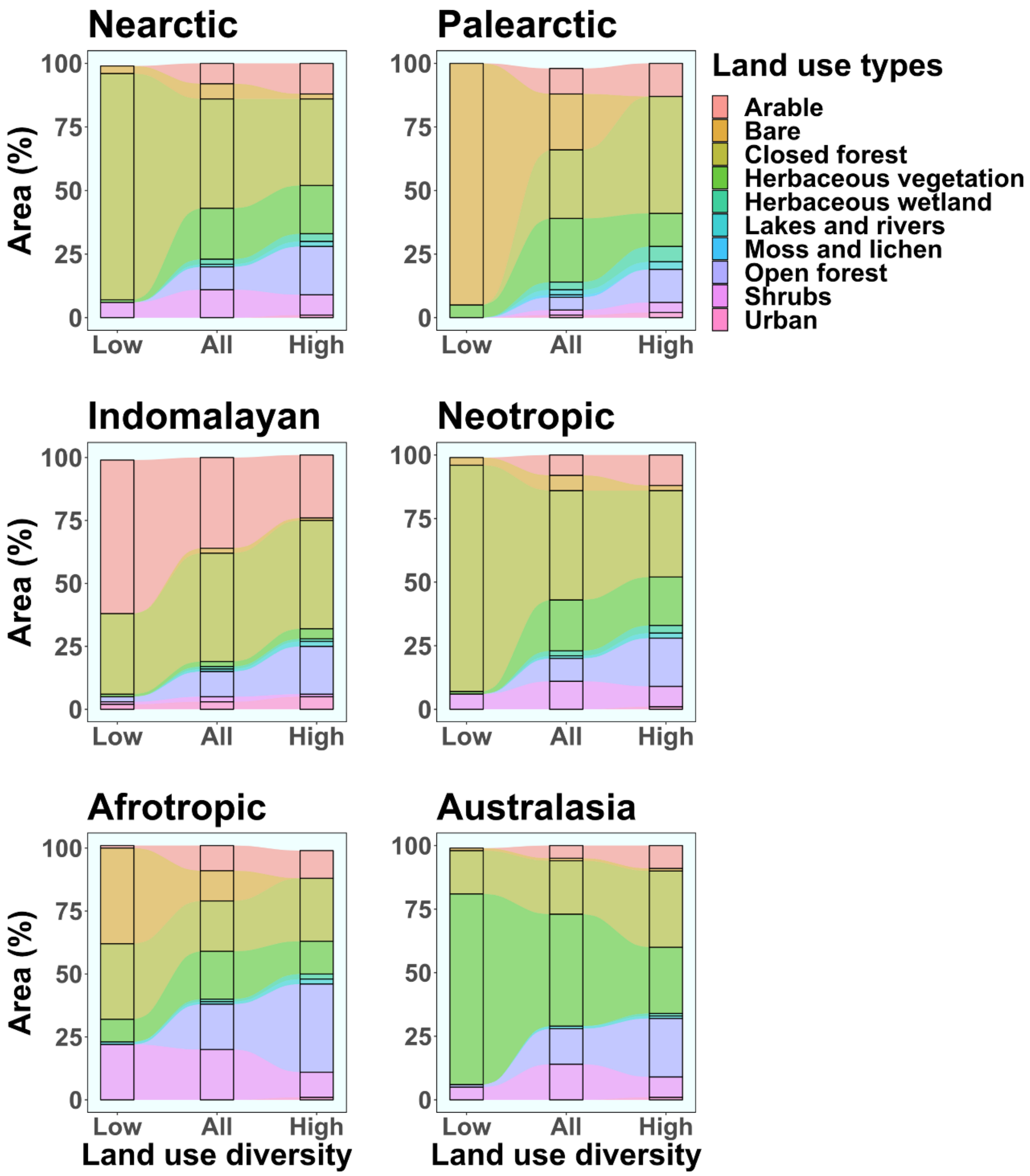

Supplementary Fig. 6. Extension of each land-use type across biogeographic realms. Contribution of each main land-use type (number of pixels) to the total area in each considered biogeographic realm. For simplicity, different types of forests were aggregated into open and closed forests.

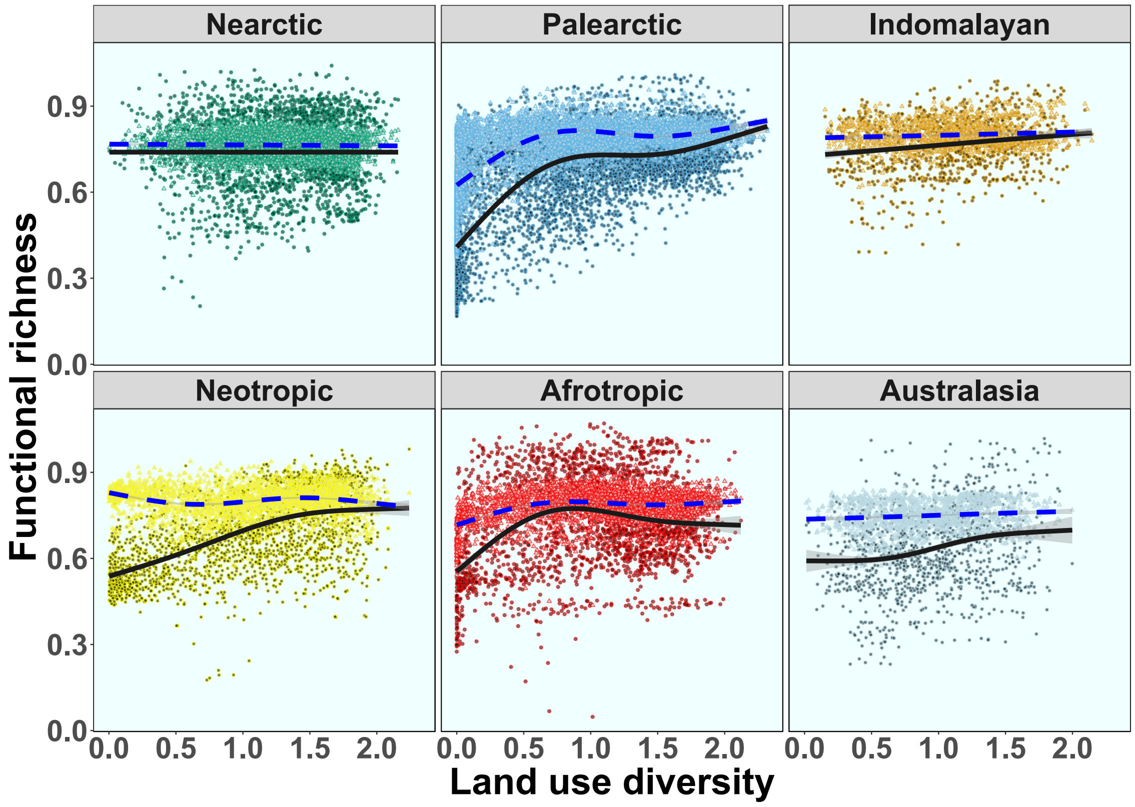

Supplementary Fig. 7. Effect of regional land-use diversity on bird richness (sensitivity analysis with smoothing factor, k = 4). Additive model fit to partial residuals, showing the effect of land-use diversity (Shannon index) on taxonomic richness (blue dashed line) and functional richness (black solid line) across the six main biogeographic realms. Each point represents a grid cell of one degree size. Taxonomic and functional richness were standardized to the maximum value observed. Nearctic: n= 2,630 grid cells. Neotropic: n = 2,262 grid cells. Palearctic: n= 6,123 grid cells. Afrotropic: n= 2,466 grid cells. Australasia: n= 1,212 grid cells. Indomalaya: n= 1,087 grid cells.

Supplementary Note 1. Why we avoid the use of Standardized Effect Sizes in this study?

Calculating standardized effect sizes (SES) or z-scores is a useful method to standardize observed measures and make them comparable across sites and gradients. In ecology, it is often used to represent how much the observed measure departs from an expected random value, if random processes governed the assemblage of communities. It is calculated by subtracting the randomly expected mean to the observed mean, and dividing this result by the standard deviation (SD) of the random values. It is widely used in ecology studies to control for the effect of richness, when using variables that strongly depend on it, such as functional richness. However, calculating SES aiming to control for species richness in studies that involve a large number of species and/or communities with very different numbers of species might be misleading. This is due to the fact that communities with a lower number of species will always tend to have SES closer to 0, because the standard deviation of the random sample will be higher. For instance, the functional richness of 10 species randomly selected from a pool of 5000 species will, due to mathematical probability, be very different from another random sample of 10 species in the same pool of species. However, if the random sample includes 1000 species, the random value will be closer to the next iteration, when another 1000 species are randomly selected. As a consequence, SD of the random values will be much higher in communities that host a low number of species, and thus, SES will tend to be closer to 0. In other words, given the same effect size (difference between the observed and the randomly expected value), communities with less species can have a much lower SES compared to communities with more species. The SES values, still represent how much the observed value departs from the randomly expected measure, but, because the randomly expected measure is highly variable in low rich communities, the SES will be closer to zero in low diversity communities.

For these reasons, in this study we decided to use different methods to assess the effect of land-use diversity on functional richness after removing the effect of taxonomic richness. Moreover, we acknowledge that taxonomic richness is a legit driver of functional richness, and often the main one. Therefore, removing its effect when looking at real-world realistic changes in functional richness means explaining only very small variations in functional richness (the ones not due to taxonomic richness).

Supplementary References

Baret, F. ; Weiss, M. ; Lacaze, R. ; Camacho, F. ; Makhmara, H. ; Pacholczyk, P. ; Smets, B. GEOV1: LAI, FAPAR Essential Climate Variables and FCover global times series capitalizing over existing products. Part1: Principles of development and production. Remote Sensing of Environment 2013, vol. 137, 299–309.

Buchhorn, M., Lesiv, M., Tsendbazar, N. E., Herold, M., Bertels, L., & Smets, B. (2020). Copernicus Global Land Cover Layers—Collection 2. Remote Sensing 2020, Vol. 12, Page 1044, 12(6), 1044. https://doi.org/10.3390/RS12061044

Cooney, C.R. et al. (2017) Mega-evolutionary dynamics of the adaptive radiation of birds. Nature, 542: 344-347.

Dinerstein, E., Olson, D., Joshi, A., Vynne, C., Burgess, N. D., Wikramanayake, E., Hahn, N., Palminteri, S., Hedao, P., Noss, R., Hansen, M., Locke, H., Ellis, E. C., Jones, B., Barber, C. V., Hayes, R., Kormos, C., Martin, V., Crist, E., … Saleem, M. (2017). An Ecoregion-Based Approach to Protecting Half the Terrestrial Realm. BioScience, 67(6), 534–545. https://doi.org/10.1093/BIOSCI/BIX014

Dunning, J.B. Jr (2007) Handbook of Avian Body Masses. Second Edition. CRC Press.

Felice, R.N., et al. (2019) Dietary niche and the evolution of cranial morphology in birds. Proceedings of the Royal Society of London B, 286: 20182677.

Fick, S. E., & Hijmans, R. J. (2017). WorldClim 2: new 1-km spatial resolution climate surfaces for global land areas. International Journal of Climatology, 37(12), 4302–4315. https://doi.org/10.1002/JOC.5086

Fitzpatrick, S (1987) Patterns of morphometric variation in birds' tails: length, shape and variability. Biological Journal of the Linnean Society, 62: 145-162.

Gorelick, N., Hancher, M., Dixon, M., Ilyushchenko, S., Thau, D., & Moore, R. (2017). Google Earth Engine: Planetary-scale geospatial analysis for everyone. Remote Sensing of Environment, 202, 18–27. https://doi.org/10.1016/J.RSE.2017.06.031

Grant, P.R. (1999) Ecology and Evolution of Darwin's Finches. Princeton Univ. Press.

Kennedy, J.D., et al. (2016) The influence of wing morphology upon the dispersal, geographical distributions and diversification of the Corvides (Aves; Passeriformes). Proceedings of the Royal Society of London B, 283: 20161922.

León-Tavares, J., Roujean, J. L., Smets, B., Wolters, E., Toté, C., & Swinnen, E. (2021). Correction of Directional Effects in VEGETATION NDVI Time-Series. Remote Sensing 2021, Vol. 13, Page 1130, 13(6), 1130. https://doi.org/10.3390/RS13061130

Magurran, A. E., & Henderson, P. A. (2003). Explaining the excess of rare species in natural species abundance distributions. Nature 2003 422:6933, 422(6933), 714–716. https://doi.org/10.1038/nature01547

Mu, H., Li, X., Wen, Y., Huang, J., Du, P., Su, W., Miao, S., & Geng, M. (2022). A global record of annual terrestrial Human Footprint dataset from 2000 to 2018. Scientific Data 2022 9:1, 9(1), 1–9. https://doi.org/10.1038/s41597-022-01284-8

Navalón, G., et al. (2019). The evolutionary relationship among beak shape, mechanical advantage, and feeding ecology in modern birds. Evolution, 73: 422-435.

Pigot, A.L., et al. (2020). Macroevolutionary convergence connects morphological form to ecological function in birds. Nature Ecology and Evolution, 4: 230-239.

Sheard, C., et al., (2020). Ecological drivers of global gradients in avian dispersal inferred from wing morphology. Nature Communications, 11: 2463.

Wilman, H., et al. (2014) EltonTraits 1.0: Species-level foraging attributes of the world's birds and mammals. Ecology, 95, 2027.
