# Supplementary material for: Land-use diversity predicts regional bird taxonomic and functional richness worldwide
Source: Nat Commun. 2023 Mar 10;14:1320. doi: 10.1038/s41467-023-37027-5 (PMC10006419; doi:10.1038/s41467-023-37027-5)
Supplement: Supplementary file 3 — Reporting Summary [file 41467_2023_37027_MOESM3_ESM.pdf]

nature portfolio

Corresponding author(s): Carlos Martínez Núñez

Last updated by author(s): Feb 2, 2023

Reporting Summary

Nature Portfolio wishes to improve the reproducibility of the work that we publish. This form provides structure for consistency and transparency in reporting. For further information on Nature Portfolio policies, see our Editorial Policies and the Editorial Policy Checklist.

Statistics

For all statistical analyses, confirm that the following items are present in the figure legend, table legend, main text, or Methods section.

n/a	Confirmed	
☐	☒	The exact sample size (*n*) for each experimental group/condition, given as a discrete number and unit of measurement
☐	☒	A statement on whether measurements were taken from distinct samples or whether the same sample was measured repeatedly
☐	☒	The statistical test(s) used AND whether they are one- or two-sided<br>*Only common tests should be described solely by name; describe more complex techniques in the Methods section.*
☐	☒	A description of all covariates tested
☐	☒	A description of any assumptions or corrections, such as tests of normality and adjustment for multiple comparisons
☐	☒	A full description of the statistical parameters including central tendency (e.g. means) or other basic estimates (e.g. regression coefficient) AND variation (e.g. standard deviation) or associated estimates of uncertainty (e.g. confidence intervals)
☐	☒	For null hypothesis testing, the test statistic (e.g. *F*, *t*, *r*) with confidence intervals, effect sizes, degrees of freedom and *P* value noted<br>*Give P values as exact values whenever suitable.*
☒	☐	For Bayesian analysis, information on the choice of priors and Markov chain Monte Carlo settings
☒	☐	For hierarchical and complex designs, identification of the appropriate level for tests and full reporting of outcomes
☐	☒	Estimates of effect sizes (e.g. Cohen's *d*, Pearson's *r*), indicating how they were calculated

Our web collection on statistics for biologists contains articles on many of the points above.

Software and code

Policy information about availability of computer code

Data collection	none
Data analysis	Code will be shared upon request (only common standard analyses were conducted).

For manuscripts utilizing custom algorithms or software that are central to the research but not yet described in published literature, software must be made available to editors and reviewers. We strongly encourage code deposition in a community repository (e.g. GitHub). See the Nature Portfolio guidelines for submitting code & software for further information.

Data

Policy information about availability of data

All manuscripts must include a data availability statement. This statement should provide the following information, where applicable:

- Accession codes, unique identifiers, or web links for publicly available datasets
- A description of any restrictions on data availability
- For clinical datasets or third party data, please ensure that the statement adheres to our policy

Data are available in Figshare (https://doi.org/10.6084/m9.figshare.21747257.v1). Data used to calculate functional and taxonomic bird richness are already publicly available in the AVONET dataset: (https://doi.org/10.1111/ele.13898). Bird distribution data by BirdLife International (http://datazone.birdlife.org/species/requestdis). Land-use diversity and NDVI values were calculated for each grid cell from Google Earth Engine (https://earthengine.google.com/); Copernicus Global Land Service website. PROBA-V Mission Exploitation (https://proba-v-mep.esa.int/proba-v-mep-toolset/geo-viewer).

Human research participants

Policy information about studies involving human research participants and Sex and Gender in Research.

Reporting on sex and gender	Does not apply to our study
Population characteristics	Does not apply to our study
Recruitment	Does not apply to our study
Ethics oversight	Does not apply to our study

Note that full information on the approval of the study protocol must also be provided in the manuscript.

Field-specific reporting

Please select the one below that is the best fit for your research. If you are not sure, read the appropriate sections before making your selection.

☐ Life sciences ☐ Behavioural & social sciences ☒ Ecological, evolutionary & environmental sciences

For a reference copy of the document with all sections, see nature.com/documents/nr-reporting-summary-flat.pdf

Ecological, evolutionary & environmental sciences study design

All studies must disclose on these points even when the disclosure is negative.

Study description	In this study, we examined the relationship between regional land-use diversity and bird taxonomic/functional richness. In order to do so, we used bird occurence data worldwide, calculated species and functional richness in each grid cell, and then ran generalized additive models using land-use diversity as the main explanatoru variable. We controlled for spatial autocorrelation and net primary productivity as possible confounding factors. We also controlled for potential different outcomes in each biogeographic realm, by including an interaction between REALM and the explanatory variables.
Research sample	We obtained spatially explicit bird distribution occurrence data from the AVONET database (https://doi.org/10.1111/ele.13898), which is based on data provided by BirdLife International (2019) about the geographic distribution of all the extant bird species (http://datazone.birdlife.org/species/requestdis). Bird worldwide distributions were extracted to an equal area grid (Behrmann projection) with a resolution of ~1 degree (~110km side cells). The datasets used represent all the extant bird species in the world, and also the six main biogeographic regions across the world at a resolution of 1 degree (~100Km2) each grid cell. Functional richness was calculated from several traits provided in AVONET. Particularly, we considered several morphological and ecological traits that are functionally important, such as: i) body mass; ii) four traits summarizing beak morphology: length from the tip to the culmen, length from the tip to the nares, beak width, and beak depth; iii) four traits reflecting body morphology: length of tarsus, wing length, tail length, and the hand-wing index; iv) diet or predominant trophic niche (categorical with ten levels; e.g., aquatic predator, insectivorous, granivorous, frugivorous, etc.); and v) primary lifestyle (categorical with five levels, e.g., aerial, terrestrial, insessorial, etc.).<br>We selected these datasets because they signify the more up to date, complete, and comprehensive datasets to date, providing accurate information about all extant bird species worldwide.accurate information about all extant bird species worldwide.
Sampling strategy	After removing grid cells with missing information, less than ten percent of land or less than five species, 15,780 grid cells were ultimately used for analyses, belonging to the six main biogeographic realms: Nearctic (2,630 grid cells), Neotropic (2,262 grid cells), Palearctic (6,123 grid cells), Afrotropic (2,466 grid cells), Australasia (1,212 grid cells), and Indomalaya (1,087 grid cells). This number of cells cover the vast majority of terrestrial (non-ice) surface on Earth. Furthermore, >99% of bird species were considered in this study, providing a worldwide coverage.
Data collection	Carlos Martínez-Núñez ensambled data from these datasets (BirdLife International (bird ocurrence), AVONET database (bird traits), using a computer with internet connection and R v.4.0.4. Ricardo Martinez-Prentice calculated land-use diversity for each grid cell, from Copernicus Global Land Service Land Cover Map (22 classes in total; Supplementary Table 2) at a 100 m resolution (CGLS-LC100), delivered from the vegetation instrument on board of the PROBA satellite (PROBA-V). We used a computer with internet connection and the cloud-based platform Google Earth Engine to collect and extract the land-use type information and calculate the Shannon diversity index of pixels in each bird grid as well as the mean NDVI. Then, these values for each grid cell were extracted to a text file.
Timing and spatial scale	We used data already collected from other studies. We downloaded data on the first week of April 2022. Especifically, bird ocurrence from BirdLife International, bird traits from the AVONET, and land-use data from Copernicus. Bird ocurrence data corresponds to the year 2019. This study covers a worldwide scale, including the six main biogeographic realms across the world.
Data exclusions	We excluded grid cells not belonging to the six main biogeographic realms (Nearctic, Palearctic, Indomalayan, Neotropic, Afrotropic and Australasian). We also excluded grid cells with less than six species because functional richness was not calculable using 5 dimensions. Finally, we also excluded grid cells that had less than 10% of land.

Reproducibility	Code and data are available for reproducibility. The response variables were: (1) bird species richness in each grid cell, and (2) functional richness (convex hull), calculated using the "fundiversity" v. 0.2.1 package in R. The main result showing that land-use diversity increases taxonomic and functional richness is reproduced across the six main biogeographic realms, with a single exception (the Nearctic reagion, with important ecological singularities that seem to explain this exception). Results seem also robust to the type of model used and the smoothing factor incorporated. Finally, the distance matrix between the species for the traits selected is strongly correlated (0.67%) with the distance matrix including only the three quantitative traits (removing the two qualitative traits). We also used the package ggplot2 v.3.3.6 to draw the plots, mgcv v.1.8-36 for fitting generalized additive models, the package ade4 v.1.7-20 to calculate trait contributions, lme4 v.1.1-27.1 to fit linear models, and visreg v.2.7.0 to calculate partial effects. Maps were drawn using the cartopy package v.0.21.1 65 in Phyton v. 3.10.0.
Randomization	Observations were not allocated in factor levels, we used the observed data. We controlled for potential confounding factors by including them as covariates (i.e., coordinates and net primary productivity).
Blinding	Blinding was not relevant to our study because we did not run any experiment. Instead, we analyzed observed data between birds assemblages and their environment.

Did the study involve field work? ☐ Yes ☒ No

Reporting for specific materials, systems and methods

We require information from authors about some types of materials, experimental systems and methods used in many studies. Here, indicate whether each material, system or method listed is relevant to your study. If you are not sure if a list item applies to your research, read the appropriate section before selecting a response.

Materials & experimental systems

n/a	Involved in the study
☒	☐ Antibodies
☒	☐ Eukaryotic cell lines
☒	☐ Palaeontology and archaeology
☒	☐ Animals and other organisms
☒	☐ Clinical data
☒	☐ Dual use research of concern

Methods

n/a	Involved in the study
☒	☐ ChIP-seq
☒	☐ Flow cytometry
☒	☐ MRI-based neuroimaging
